# What's the catch? Validity of whaling data for Japanese catches of sperm whales in the North Pacific

**DOI:** 10.1098/rsos.150177

**Published:** 2015-07-15

**Authors:** Yulia V. Ivashchenko, Phillip J. Clapham

**Affiliations:** National Marine Mammal Laboratory, Alaska Fisheries Science Center, 7600 Sand Point Way NE, Seattle, WA 98115, USA

**Keywords:** whaling, illegal whaling, North Pacific, sperm whale, Japan, USSR

## Abstract

The failure of international efforts to manage commercial whaling was exemplified by revelations of large-scale illegal whale catches by the USSR over a 30 year period following World War II. Falsifications of catch data have also been reported for Japanese coastal whaling, but to date there has been no investigation of the reliability of catch statistics for Japanese pelagic (factory fleet) whaling operations. Here, we use data of known reliability from Soviet whaling industry reports to show that body lengths reported to the International Whaling Commission (IWC) by Japanese factory fleets for female sperm whales caught in the North Pacific are not credible. In 1968/1969, Japanese whaling fleets in the North Pacific killed 1568 females, of which 1525 (97.3%) were reported as being at or above the IWC's minimum length of 11.6 m (legal-sized females, LSFs). By contrast, Soviet fleets operating during this period killed 12 578 females; only 824 (6.6%) were LSFs. Adjusting for effort, catches of LSFs were up to 9.1 times higher for Japan compared with the USSR, and even higher for very large females. Dramatic differences in body length statistics were evident when both nations operated in the same area. Significantly, the frequency of LSFs and very large females in the Japanese catch markedly declined after the IWC's International Observer Scheme in 1972 made illegal whaling more difficult. We conclude that the Japanese length data reflect systematic falsification of catch statistics submitted to the IWC, with serious implications for the reliability of data used in current population assessments. The apparent ease with which catch data were falsified in the past underscores the necessity of transparent and independent inspection procedures in any future commercial whaling.

## 1. Introduction

Since 1946, commercial whaling has been managed under the International Convention for the Regulation of Whaling, which created the International Whaling Commission (IWC) to oversee research, set catch and minimum size limits, establish whaling seasons and closed areas, and to serve as a repository for the catch statistics that all member states are required to report. Yet beginning in 1948, the USSR began a 30 year global campaign of illegal whaling that made an estimated 178 811 unreported catches [1,2]. The true Soviet catch record has now been largely reconstructed using data of known reliability from internal whaling industry reports that were secret until their declassification in the 1990s. This major deception was possible because whaling was not subject to independent inspection until the introduction by the IWC of an International Observer Scheme (IOS) in 1972. Until then, there was no way to verify the accuracy of catch reports, and even after 1972 it is known that international observers failed to report some infractions [3], through either distraction, direct collusion, or the fact that a single inspector could not be present on deck 24 h a day.

One species that suffered disproportionately in these illegal catches was the sperm whale, *Physeter macrocephalus*. Sperm whales are characterized by strong male-biased sexual size dimorphism and are a globally distributed species that was the target of extensive commercial whaling beginning in the late 1700s. More than 760 000 were killed in the twentieth century alone [4]; of these, almost 315 000 were taken in the North Pacific, the great majority by Japan and the USSR. Extensive catches were made from shore whaling stations in northern Japan and the Kuril Islands [5]. Pelagic catches of this species from Japanese whaling factory fleets began in 1954, with the USSR following in 1960. Pelagic catches rapidly increased beginning in 1963, and during the peak years of 1965–1970 a total of 89 462 sperm whales were caught, including an annual maximum of more than 18 000 in 1966 [5,6].

As part of its illegal whaling campaign, the USSR routinely ignored whaling regulations, killing male and female sperm whales below the IWC's minimum length limit of 11.6 m [2]. In official reports to the IWC, the Soviets falsified female lengths, or misreported illegal-sized females as larger males in order to make the catch figures consistent with reported oil production (because adult male sperm whales are much larger than females, and thus yielded more oil, it was more believable to report as a male an undersized whale whose length had been intentionally increased). However, the true data on the numbers and biological characteristics (sex, length, etc.) of the catches were preserved in secret internal reports that were declassified only after the collapse of the USSR [2,7].

Similar systematic falsification of catch data for sperm whales and other species was subsequently reported for coastal whaling stations in Japan, a practice which apparently continued after the IWC's whaling moratorium came into effect in 1985 [8–10]. It has been also been stated that Japanese catches of baleen whales were significantly under-reported owing to a system in which catcher boats had individual quotas and were paid a bonus for the largest whales; because of this system, reportedly many whales could be killed but only the largest were delivered for processing, with others not reported in official statistics [11].

Despite these concerns, there has to date been no investigation of the reliability of the officially reported catch statistics for Japanese pelagic (factory fleet) whaling, although Japanese members of an IWC working group stated in 1999 that ‘Japanese pelagic catch numbers are correct’ [12]. In 1983, an analysis of length data for Japanese pelagic catches of sperm whales in the northwestern North Pacific raised questions about the reliability of this information [13], but at the time there was no way to independently assess whether the catch data were likely to be accurate. Here, using comparisons of data of known reliability from Soviet whaling industry reports, we conclude that the length distributions reported by Japan to the IWC for catches of female sperm whales in the North Pacific are not credible, and indicate extensive and intentional misreporting of whaling catch data from this ocean.

## 2. Material and methods

Catch data reported to the IWC by the USSR for whaling operations in the North Pacific were routinely falsified. However, Ivashchenko *et al*. [14] used information in formerly secret internal Soviet whaling industry reports to correct the catch record for all species (including sperm whales) taken in this ocean. These reports were found and copied in the State Archive of the Primorskiy Region in Vladivostok, Russia. The source material includes: (i) scientific reports summarizing catches by area and time, as well as measurement and biological data, and assessments of the status of species and stocks; (ii) whaling production reports, which summarize the types and quantities of products derived from the caught whales; and (iii) reports from the Soviet government's official whaling inspectors who were present aboard factory ships. These materials were previously unpublished and were largely unavailable until their declassification. That the data in these reports are reliable has been confirmed by Russian biologists who worked on Soviet factory fleets [7].^1^ However, the reports contain varying amounts of information, and locations and other details of catches are not available for all years. Details of the reports are summarized elsewhere [14]. A summary of Soviet catches of sperm whales in the North Pacific was recently published [5].

In this study, we used data from the ‘true’ Soviet reports to assess the reliability of the official Japanese catch statistics as compiled in the IWC's catch database [6]; this large dataset includes information that whalers were required to report regarding individual catch dates and locations as well as biological information such as sex and length. We focused on the years 1968–1969 and 1973–1974: these were years for which sperm whale length data of known reliability were available for the USSR; this was not the case for 1970–1972. The two periods bracket 1972, the year in which the IWC's IOS was introduced [17]; at this time, independent inspectors began working on factory ships, thus (in theory) reducing or eliminating illegal catches.

For the two data sources, we examined the number of killed female sperm whales whose length was equal to or greater than 11.6 m, the IWC's minimum allowable length for catches of this species (referred to here as legal-sized females, LSFs). Japanese length data for all years, and lengths for the USSR for 1973–1974, were given in individual catch records in the IWC's catch database (Soviet catch and length data for those years were identical to those found in the Soviet reports). For the Soviet internal report data for 1968 and 1969, lengths were given in binned length categories, usually in half-metre values (e.g. 9.1–9.5 m, 9.6–10 m, etc.). Because one of these categories included lengths above and below 11.6 m (11.5–11.7, we assigned a proportion of animals in this category to the 11.6+m group. For example, for the *Dalniy Vostok* and *Slava* joint report for 1968, two-thirds (271) of the 410 females in the 11.5–11.7 m bin were assumed to be at least 11.6 m long. Although this apportionment is somewhat arbitrary, it made little difference to the analysis of the large overall sample. The original binned data are shown in translations of the report tables in the electronic supplementary material.

To factor in catch effort, we calculated the number of days on which each whaling fleet caught sperm whales, and multiplied that by the number of catcher boats operating in each fleet to give a measure of catcher work days (CD). The number of catchers was 14 for all Soviet fleets, but in Japanese fleets varied from 7 to 9 per fleet; calculations of CD for Japanese fleets are shown in table 1. We then compared the mean daily catch per catcher (MDC=LSF/CD) for the two nations.

**Table 1.** Number of catchers (*C*), days catching sperm whales (*D*) and catcher work days (CD=*C*×*D*), for Japanese factory ships operating in the North Pacific.

	factory fleets															
	Tonan Maru			Nishin Maru			Tonan Maru 2			Kyokuyo Maru 3			Nishin Maru 3			
year	*C*	*D*	CD	*C*	*D*	CD	*C*	*D*	CD	*C*	*D*	CD	*C*	*D*	CD	total CD
1968	9	45	405										8	84	672	1077
1969	9	54	486	9	56	504				9	56	504				1494
1973							8	30	240	7	29	203	7	38	266	709
1974							7	27	189	7	32	224	7	31	217	630

We hypothesized that if female length data were misreported by Japan prior to the IOS, then the occurrence of female sperm whales measuring at or above the pre-1972 minimum legal length^2^ should be markedly lower when catches were independently inspected in 1973/1974.

## 3. Results

Catches of sperm whales in the North Pacific increased dramatically beginning in 1963. Peak catches occurred in 1966, with 15 205 and 3000 taken by the USSR and Japan, respectively [5,6]. In 1968 and 1969, Japanese whaling fleets operating across the North Pacific reported killing 1568 female sperm whales, of which 1525 (97.3%) were reported as being at or above the minimum legal length of 11.6 m. By contrast, Soviet fleets operating during the same time period killed 12 578 females, only 824 (6.6%) of which were ≥11.6 m. This difference is extremely significant (*χ*^2^_1_=3832.5 (*p*<0.0001)). Statistical differences in the frequency distribution of the Japanese and Soviet catches for the years 1968/1969 combined were assessed with a Kolmogorov–Smirnov test at the critical level of *α*≤0.01 [18]. The difference was statistically significant (*D*=0.488, *p*<0.01).

The proportion of LSFs in the Japanese catch was thus almost 15 times as large as that of the USSR; however, a better index of the difference in the female catches involves figures that take into account catcher effort. Comparisons of MDC data (the mean daily catch, per catcher, of LSFs) are shown in table 2. Despite much larger catches and greater catch effort by the Soviet whalers, Japanese fleets reported taking 7.6 times as many LSFs in 1968, and 9.1 times as many in 1969.^3^

**Table 2.** Sperm whales killed by the USSR and Japan, 1968–1969 and 1973–1974. (LSFs (those with lengths ≥11.6 m). CD, catcher days (from table 1 for Japan, and based on 14 catchers for all Soviet fleets). MDC=LSF/CD, the mean daily catch (per catcher) of LSFs. The last column is the ratio of MDC values, Japan : USSR. *χ*^2^-comparisons for females and LSFs between the two countries' fleets were as follows: 1968 χ^2^_1_=1795.9 (*p*<0.0001); 1969 χ^2^_1_=1943 (*p*<0.0001).)

		sperm whale catches					
year	nation	males	females	LSF	CD	MDC	MDC ratio
1968	USSR	4642	6898	443	6062	0.07	×7.6
	Japan	2416	584	573	1077	0.53	
1969	USSR	3734	5680	381	5390	0.07	×9.1
	Japan	2016	984	952	1494	0.64	
1973	USSR	2043	2285	62	2674	0.02	×3.5
	Japan	1443	360	49	709	0.07	
1974	USSR	1884	2081	54	2884	0.02	×3.5
	Japan	1342	461	47	630	0.07	
total	both	19 520	19 333				

The year 1969 stands out because of the remarkably high numbers of LSFs (952) reported by Japan. Furthermore, many females were reported as being of particularly large size: 141 females caught in 1969 had reported lengths of 12.5 m or more (maximum length 14 m); by contrast, in the same year the USSR killed 5680 females, of which only two exceeded this length.

The maximum Japanese catch of LSFs in a single day was 115, reportedly taken (by three fleets operating in two widely separated areas) on 11 August 1969. This is particularly difficult to accept given that in August 1969 two Japanese factory fleets were catching in an area that had already been worked (and presumably depleted to some extent) by three Soviet whaling fleets. Indeed, the contrast between catch numbers in the two nations' operations in this area is sufficiently marked to be worth describing in detail.

**Figure 1.**
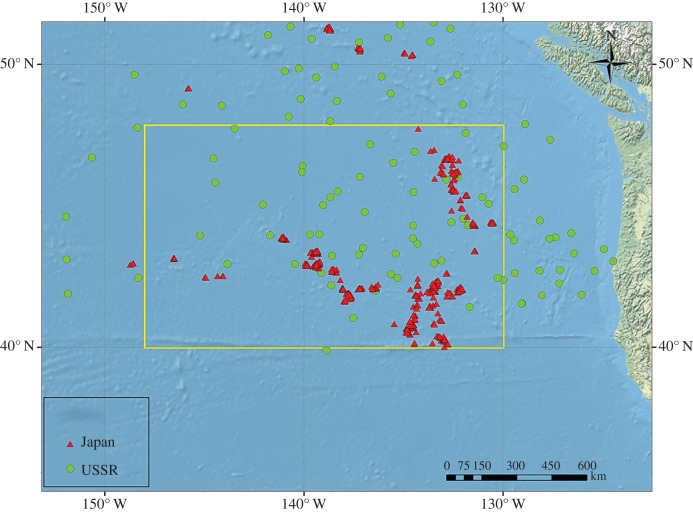
Locations of Soviet and Japanese sperm whale catches in the eastern North Pacific, 4–20 August 1969 (yellow box). Each point represents anywhere from one to many catches.

On 14 separate days from 4 to 20 August 1969, two Japanese factory fleets caught sperm whales in the eastern North Pacific, operating between 40 and 48 N, and longitudes 130 and 149 W (the southern portion of an area defined by the USSR as the ‘Eastern Region’, figure 1). During this time, they caught 505 males and 461 females; of the latter, 454 (98.5%) were reported as being of legal size.^4^ Three Soviet fleets worked this same area for various periods, as follows: *Slava* (10 May–20 August), *Dalniy Vostok* (17–28 July) and *Vladivostok* (1 June–10 July). Together, the three fleets killed 3650 sperm whales, including 2180 females; of the latter, only 80 (3.7%) were of legal size. More detailed records are available for *Slava*, which was still operating in the region when the Japanese fleets were there: from 1 to 20 August, *Slava* killed 438 sperm whales, of which 188 were males. Of the 250 females taken, only 8 (3.2%) were LSFs; the corresponding Japanese figure is thus more than 30 times this percentage.

The maximum Japanese single-day catch of LSFs in 1968 was 44 (on 14 July), when only two fleets were working on sperm whales. This dropped to seven and six for 1973 (2 August) and 1974 (7 June), respectively; in both years, three fleets were hunting this species.

Because introduction of the IOS in 1972 made illegal whaling much more difficult, we predicted that the frequency of large females in the Japanese catch data should markedly decrease in subsequent years, and be more similar to the numbers taken by the USSR. This was the case (table 2): the Japanese catch of LSFs had declined to 3.5 times that of the Soviets and, in contrast to 1968/1969, there were only two females reported with lengths ≥12.5 m.

**Figure 2.**
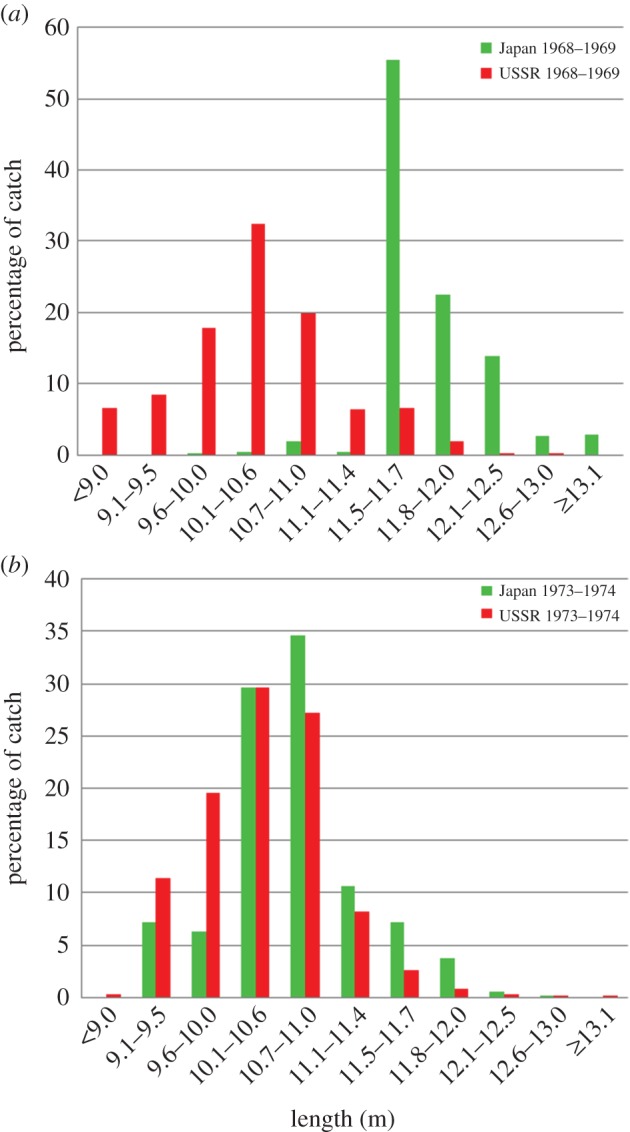
Length frequency distributions (percentage of total female catch) for Japanese and Soviet catches of female sperm whales in (*a*) 1968/1969 and (*b*) 1973/1974.

Length frequency distributions for Japanese and Soviet catches of female sperm whales in 1968/1969 and 1973/1974 (before and after introduction of the IOS) are shown in figure 2.

## 4. Discussion

Sperm whales were by far the biggest target for commercial whaling in the North Pacific, with almost 315 000 killed (primarily by Japan and the USSR) during the twentieth century [4,5]. The major misreporting of catch data by the USSR has been known for many years, and similar falsifications (albeit on a smaller scale overall) have previously been reported for Japanese coastal whaling operations [8–10]. Disclosure of the latter was possible only because the true catch statistics had been preserved by retired whaling company staff and in the records of biologists; no such material has been forthcoming for Japanese factory fleet operations, although as noted above the reliability of length data for some sperm whale catches in the northwestern Pacific was discussed at the IWC in the early 1980s [12].

Our comparisons of reported Japanese body length statistics with the Soviet catch data for female sperm whales in the North Pacific highlight that the former are not credible, specifically in terms of the high frequency with which LSFs were caught, and of the marked decline in catches of such animals after implementation of the IOS brought independent observers aboard factory ships. We suspect that the large catches made prior to the IOS actually involved numerous undersized whales; that almost 100% of the males killed in 1968 and 1969 were also reported to be above the minimum size limit suggests that it was not only females involved in the misreporting. Mature male sperm whales are much larger than females; however, the family groups found in lower latitudes typically contain much smaller immature males, and it further strains credibility to accept that almost every animal caught was of legal length [5].

In this regard, it is important to distinguish between the practice of occasional ‘whale stretching’, a well-known practice throughout the whaling industry whereby the reported length of an undersized animal killed unintentionally was increased by a small amount to avoid an infraction, and extensive intentional catches of undersized whales, with systematic falsification of the associated length data. The latter is well known to have been characteristic of Soviet illegal whaling, and the analyses reported here strongly suggest that a similar practice was followed by Japanese pelagic whaling fleets.

The Japanese statistics for 1969 are particularly anomalous, with more LSFs reported killed (454) in only 14 days than three Soviet fleets managed to take (381) during the entire year. Indeed, our analysis of catch statistics for Soviet and Japanese whaling fleets operating in the same region in August 1969 highlights the implausibility of the Japanese catch records, which claimed that almost all of the 461 females caught at this time were legal, compared to only 3% of the Soviet catch. That the Japanese could find so many large females in such a short period, and after Soviet fleets had already swept the area, is simply not credible, particularly in view of a statement which appears in the Soviet joint scientific report for the *Slava* and *Dalniy Vostok* fleets [19], p. 6:

During the whole period of work in the Eastern Region in 1969 there were constant difficulties with whaling conditions. There were many days with no catches and long daily transits to find groups of whales that in this year were widely spread out and very skittish… In the second half of August, because of the decline in daily catches, the fleet was forced to leave the Eastern Region on 20 August and move west.

The high frequency of LSFs, and of very large females, in the Japanese catch statistics cannot be explained by differing size selectivity or greater catcher efficiency relative to Soviet whaling operations. Soviet whalers, who were required to meet often high production targets, actively sought out the largest animals because of their high production value [7] (N. Doroshenko 2006, personal communication). By the mid-1960s, Soviet factory fleets were highly experienced in the business of whaling, and in some Antarctic operations deployed more than 20 catcher boats per fleet. Globally, the huge illegal catches are testament to the efficiency of the Soviet whaling fleets; as examples, two fleets killed almost 25 000 humpback whales (*Megaptera novaeangliae*) in just two seasons in the Antarctic [20], and Soviet whalers are believed to have wiped out the bulk of the right whale (*Eubalaena japonica*) population in the eastern North Pacific [21].

Given confirmed falsifications of catch data in the Japanese coastal fishery, it should not be surprising that such infractions also occurred in pelagic operations. Additional indirect evidence that data falsification was occurring comes from the fact that Japanese biologists (who were not present on all factory ships) were not permitted to measure whales (T. Kasuya 2015, personal communication). Furthermore, there appear to have been repeated attempts to actively obstruct inspection at least in the coastal fishery, and in this context we note the following statement by Tillman (unpublished data):^5^

As the last supervisor of the US observer program during 1976–86, I can also attest to the uncooperative attitude of the Japanese Government and to the complete failure of the whaling industry to ensure that my two observers were on site to perform their duties where the catches were landed. The litany of perfidy ranged from sending them to the wrong land station hundreds of miles away to outright lying about when the catches were coming in when they got to the correct station. These problems did not arise because of language barriers since they both were Japanese-American citizens who spoke Japanese fluently and had Master of Science degrees as biologists. They knew what they were doing, were motivated to do well, and had to use considerable ingenuity to outwit the whalers. Nonetheless, they were lucky to observe even 50 percent of the landings in any given season.

Taken together, the evidence strongly indicates extensive illegal catches, and systematic falsification of associated length data, in both coastal and pelagic Japanese whaling operations. This has serious implications for population assessments, which rely upon an accurate catch record to assess the degree of recovery of current stocks relative to their pre-whaling abundance.

The Soviet methods for falsifying catch data in reports to the IWC, in addition to misreporting numbers, included increasing the length of animals, reporting smaller females as larger males (see above), or ‘converting’ two or three small whales and reporting them as one or two larger whales; this was necessary in order for the numbers and lengths of the catches to be consistent with reported figures for production of oil or other products [2,7]. It is not known how Japanese whalers treated catch statistics, but the unusually high frequency of LSFs suggests that lengths of undersized animals were artificially increased to meet or exceed the legal minimum. A Japanese biologist who worked on factory ships in the North Pacific and the Antarctic believes that catch numbers and sex were probably not changed, ‘but length and reproductive status of females certainly were’ (T. Kasuya 2015, personal communication). However, simply increasing the length of small whales would not be sufficient to match production totals, and it is not clear how this problem was resolved.

Because length data are not available for Soviet fleets for all years, we are unable to assess the extent to which Japanese falsifications occurred outside the period studied here. However, only 50 females were reported as being caught by Japan for the years 1963–1965 combined; at this time, the fleets were hunting farther north and concentrating on the males that predominated in those latitudes. In 1966, Japanese catch effort began to shift to the south, into regions where females and family groups are commonly found [5]; catches of females for 1966 and 1967 were 166 and 335, respectively, with most reported as being of legal size [6]. We do not know if falsification of data began prior to 1968, but whatever the case, we suggest that an assessment of Japanese catch statistics for baleen whales (those subject to catch or length restrictions), and of sperm whales in other oceans, is warranted. In some cases, additional Soviet data are available for such comparisons.

The frequency, and apparent ease, with which illegal catches were made in the Soviet and the Japanese fishery supports the idea that, in the absence of adequate inspection procedures, large-scale cheating may prove too great a temptation to resist; this problem is not unique to whaling, and has afflicted various other commercially valuable fisheries [2,22]. Overall, the occurrence of illegal catches of sperm and other whales emphasizes the need for a truly transparent and independent observer scheme in any future commercial whaling operations.

## Supplementary Material

Single file (Ivashchenko & Clapham supplementary material) giving translation and original Russian material.

## Footnotes

1 The North Pacific report data are identical in nature to the true data on the more extensive illegal Soviet catches in the Southern Hemisphere, which were officially accepted by the IWC at the 47th meeting of the Scientific Committee in 1996 (‘The Committee agreed that revised Soviet … catches should be incorporated into the IWC databases’. [15], p. 80). The Scientific Committee also noted that the ‘authenticity of the [Southern Hemisphere catch] records was supported by the personal experiences of Zemsky, Berzin, Tormosov and Mikhaliev, each of who had participated in Antarctic expeditions and headed laboratories for some 15–30 years’ [15], p. 82. Furthermore, at its annual meeting in May 2015, the Scientific Committee, with regard to the true North Pacific data collected by us, recommended ‘that the revised data for all species be entered into the IWC catch database as a matter of priority and that the false data originally submitted … for these expeditions be removed’. [16].

2 The minimum length was reduced by IWC to 9.2 m in 1972; thus, smaller females were legal to catch after this date. However, because we wanted to investigate potentially illegal catches prior to implementation of the IOS, our analysis focused on the occurrence of 11.6+m females before *and* after the introduction of inspection. Consequently, the term ‘legal-sized females’ here refers to the legal minimum length prior to 1972.

3 We considered comparing the Japanese data against the official (falsified) catch statistics reported to the IWC by the USSR to see whether they were similar. However, the two datasets are not comparable because the USSR changed the sex of the majority of the female catches: in 1969, they reported taking 7423 males and 775 females, but the true totals (table 2) were 3734 and 5680, respectively.

4 Of the 505 males, 501 (99.2%) were also reported as =11.6 m. Overall, Japan reported killing 2016 males in 1969, with 2008 of these (99.6%) stated as being of legal length. The equivalent catch figures for 1968 were 2416 males, of which 2412 (99.8%) were reported as being equal to or greater than 11.6 m in length.

5 M. F. Tillman 2007, The scientific and technical underpinnings of the moratorium on commercial whaling. Background paper contributed to the Pew Symposium on the State of Conservation of Whales in the 21st Century, April 2007, New York, 12 p. (Available from author.)

## Data accessibility

The data on Japanese sperm whale catches were taken from the IWC's Catch Database (v. 5.3, 25 October 2012) [6], available from the IWC, Cambridge, UK. An English translation of key pages used in the analysis is provided in the electronic supplementary material.
