# Supplementary material for: What's the catch? Validity of whaling data for Japanese catches of sperm whales in the North Pacific
Source: R Soc Open Sci. 2015 Jul 15;2(7):150177. doi: 10.1098/rsos.150177 (PMC4632589; doi:10.1098/rsos.150177)
Supplement: Single file (Ivashchenko & Clapham supplementary material) giving translation and original Russian material. [file rsos150177supp.pdf]

SUPPLEMENTARY MATERIAL:
TRANSLATION FROM THE RUSSIAN OF KEY PORTIONS OF SOURCE MATERIALS

[The following are English translations of pages and tables from internal Soviet whaling reports, as used in this analysis. The entire page is translated for each part, but the relevant sections providing key information for the analyses are highlighted in yellow. The matching Russian originals are linked to the translations by ITEM number, below and in the title of each image.]

ITEM 01: *Dalniy Vostok* & *Slava* whaling fleets joint Scientific Report for 1968, page 4:

...showed the absence of whale aggregations; after that, on May 16, the fleet [*Vladivostok*] headed in a southeasterly direction with the goal of continuing whaling in the Eastern Region, working aggregations of family groups of sperm whales. In the Eastern Region the fleet worked from May 19 through August 25, while catching only small sperm whales, mainly females. While approaching the North American coast the fleet had a small number of baleen whales in the catch. The fleet was working in this area for almost half of its operating time and caught more than 50% of the total number of whales there [for the season].

Nevertheless, by the end of August daily catches began to decline, and this, together with unfavorable weather conditions, was the reason the fleet had to leave the Eastern Region. Starting on August 26 through September 7 the fleet was moving in a westerly direction, towards the Kuril Islands, where it worked until November 14, e.g. through the end of the whaling season. During the period of time that *Slava* worked in the Kurils region (from Sangar Strait to the middle Kurils), the majority of the catch consisted of family groups, mainly females, and only in Diana and Vreis Straits were large males caught over a few days. Baleen whales were rare and consisted mainly of sei whales and single fin whales.

Thus, during the whole 1968 whaling season, the *Slava* whaling fleet worked mainly in two areas: the Eastern Region for 99 days, and the Kurils for 79 days, with catches of 3,711 and 1,484 whales, respectively.

In the Central Region, where the fleet worked for only a short time and during transits, they killed 185 whales.

The total catch for the *Slava* whaling fleet during the 1968 season was 5,380 whales, including 5,139 (95.5%) sperm whales, sei....

ITEM 02: *Dalniy Vostok* & *Slava* whaling fleets joint Scientific Report for 1968, page 6:

...aggregation of whales, the fleet [*Dalniy Vostok*] went further south and worked in the Central Region during 6-30 October, on the same concentrations of whales that it worked in August. The *Dalniy Vostok* whaling fleet finished its whaling season on November 10 in the area around the central Kurils.

During the 1968 season the *Dalniy Vostok* whaling fleet was mainly working in the following regions: Eastern Region for 71 days, with a catch of 2,277 whales; Central Region for 46 days, with a catch of 1,829 whales; Aleutian Islands for 23 days, with a catch of 302 whales, and the Commander Islands for 9 days and 134 whales. Around the Kurils, where the fleet worked for only a short time and during transits, a total of 160 whales were caught.

The total catch of the *Dalniy Vostok* whaling fleet during the 1968 season was 5,378 whales, which included: sperm whales 4,853 (90.23%), fin 297 (5.5%), sei 154 (2.8%), blue 28 (0.5%), humpback 24 (0.49%), and Brydes 22 (0.48%).

Information about joint catches by the fleets *Dalniy Vostok* and *Slava* are shown in the tables.

The attached maps show the tracks of the fleets, and catches of whales by species and areas.

Legend for the maps:

____ *Slava* track
------ *Dalniy Vostok* track

Sperm whales ▲ 100-500 whales ▲ 50-100 ▴ <50
• blue whale ☐ sei whales + Brydes
⊙ fin whale ▵ humpback whale

ITEMS 03-06: *Dalniy Vostok* & *Slava* whaling fleets joint Scientific Report for 1969, pages 5-8:

[Page 5]

Data analysis was conducted by all the scientists noted above. In order to collect more complete data on population structure, distribution and other questions, in the 1969 season, as in the previous seasons, results of the studies collected on the two whaling fleets (*Slava* and *Dalniy Vostok*) were combined in the analysis.

The current report was written by the senior scientists from both factory ships, V.L. Vladimirov and A.A. Kuzmin, and by junior scientists A.N. Nikolaeva and V.I. Privalikhin.

Sections on the introduction, whaling conditions, characteristics and status of populations for commercial species of whales in the Central Region were written by A.A. Kuzmin.

The section on characteristics of populations of commercial species of whales in the Eastern Region and the conclusions section were written by V.L. Vladimirov.

The section describing results of the whaling season and the condition of the whaling resources in the northern part of the Pacific Ocean for the season was written by V.I. Privalikhin.

Whaling conditions

The main whaling areas in the North Pacific in 1969, as in 1968, were three regions:

1. Eastern Region: 40°-50° N 129°-160° W
2. Central Region: 40°-50° N 160° W-160° E
3. Kurils-Japan: 34°-47° 20' N 159°-173° E

The *Slava* whaling fleet began its work on April 19 in the area with coordinates 42° 22'-46° 24'N 149° 23' - 159° 48'E. It worked in this area until April 24, catching mainly sperm whales. The total...

[page 6]

...catch was 89 sperm whales and 3 fin whales. From 25 April through 9 May the fleets worked in the Central Region (40°10' - 46° 17', 163° 35' - 161° 58'W), where they killed 313 whales: 292 sperm, 11 fin, 6 blue and 4 sei whales. However, 15 days of work in that area showed that it was not reasonable to continue whaling there, and the fleet left the area on May 10 with the goal of continuing whaling in the Eastern Region on aggregations of family groups [of sperm whales].

From May 10 through August 20 the *Slava* whaling fleet worked in the traditional area (Eastern Region), mainly on sperm whales. During the whole period the total catch was 2,379 (2,120 sperm whales and 259 baleen whales). In the northern and northeastern sections of this region the catch mainly consisted of single males and groups of male sperm whales. In the central and eastern parts of the region (closer to the southern boundary) the fleet worked on family groups (females and under-sized males). Fin whales (95) were mainly caught in the western and northwestern sections, blue whales (19) in the western and central parts, and sei whales (142) in the western and central parts of the region. Humpback whales (4) were found in the eastern section.

Unlike during the previous year, this year was marked with a later arrival (end of June - beginning of July) of sperm whale family groups. The possible reason for this was less favorable hydrology and weather conditions (the average temperature of the air and water (at a depth of 8 meters) in June was lower than last year, with very few sunny days). During the whole period of work in the Eastern Region in 1969 there were constant difficulties with whaling conditions. There were many days with no catches and long daily transits to find groups of whales that in this year were widely spread out and very skittish.

[page 7]
Whalers, as a rule, conducted whaling in a group: once whales were found, 8-10 catchers began the hunt using a pen method, sometimes killing all whales including those 7 meters long. This intensity of catches was brought about by very high, non-scientifically-based production plan targets. Despite all the conclusions and recommendations from the biological science groups of the whaling fleets in 1968 the season to lower the target plan down to 900 thousand centners [a Russian measure of weight] a year, which was approved by the Scientific Council of TINRO, the target plan for 1969 and planned 1970 was still set at twice this number.

Intensive whaling on the aggregations of whales in the Eastern Region was dramatically reflected in the abundance and biological status of the American population of sperm whales. The annual declines in sperm whale abundance in this area created especially difficult whaling conditions during the 1969 season.

In the second half of August, because of the decline in daily catches, the fleet was forced to leave the Eastern Region on 20 August and move west.

During the period from August 21 through September 5, the fleet *Slava* worked in the western section of the Central Region catching large sperm whales and a few baleen whales. During that time they caught 48 sperm, 3 fin and 11 sei whales. With a large deficit in attempts to meet yearly and quarterly targets, the fleet could not continue its work in the Central Region due to an unstable daily catch, and moved to the Kurils region to work on family groups of sperm whales.

[Page 8]
Starting on September 6 and through the end of the season the *Slava* whaling fleet worked in the area defined by coordinates 47-34 N and 159-143 E. During the whole period working in the Kurils-Japan region (from the eastern coast of Honshu Island through the central part of the Kuril Islands) the *Slava* fleet was mainly catching family groups of sperm whales, mainly females. Large sperm whales were found rarely. Based on the experience of previous years a scout vessel was sent in September to search Vries and Diana Straits. This year no whales were found in the Straits. During the 1969 season, in waters around the cental and southern Kurils, only widely distributed groups of sperm whales were found.

Only 30% of the Target Plan for September was fulfilled. The majority of whales around the Kurils-Japan region were killed in October-November, east of Honshu and Tsugaru Strait (10-500 miles).

During the entire 1969 whaling season, the *Slava* fleet worked mainly in two regions: Eastern for 101 days and Kurils for 76 days; the catch in each area was 2,379 and 1,248 whales [respectively].

In the Central Region, where the fleet worked for 28 days, they caught 375 whales.

The total catch for the *Slava* whaling fleet in the 1969 season was 4,002 whales with: sperm whales 3,704, fin 113, sei 157, blue 25, and humpback 3.

The *Dalniy Vostok* whaling fleet began its whaling season on July 17 in the Eastern Region (46 17' N 141 20' W). In the beginning of the season the fleet worked on family groups of sperm whales, catching primarily females. Following the decision made by the Whaling Council, on July 19 the fleet with all its catchers left towards the area with the coordinates...

ITEM 07: *Dalniy Vostok* & *Slava* whaling fleets joint Scientific Report for 1969, page 10:

In the first half of October the *Dalniy Vostok* fleet conducted whaling in the southern part of the Central Region inside the area within latitudes 31-36 N and longitudes 170 W-170 E. Whaling was based on family groups [of sperm whales] with limited choice. Small numbers of Bryde's whales were caught. The majority of them were found in the area of Emperor Ridge at 34-35 N. By the end of October, due to declining weather conditions and fulfilment of the yearly Plan Target, the fleet left this area with the goal to continue whaling in the waters around the Kuril Islands, where the fleet worked until the end of the season. During October 58.4% of the fleet's catch consisted of illegal sperm whales.

Despite meeting the yearly Plan Target by the end of October, by the order of the Whaling Fleets Administration the fleet was instructed to continue whaling until November 7. In November the fleet worked around the Kuril Islands. Groups of small sperm whales were seen from the oceanic side but no hunting was conducted. In 7 days in November a total of 7 sperm whales were caught, of which 4 were small-sized females.

During the whole whaling season (from July 17 through November 6) the *Dalniy Vostok* fleet caught 1,756 whales. The main whaling area was the Central Region between longitudes 160 W and 160 E, with the southern boundary at 31 N, with the majority of whales found in the central and southern parts of this region.

During the 1969 season the factory ship *Dalniy Vostok* processed 8 fin, 7 blue, 25 sei and 95 Bryde's whales.

During 1969 season the *Slava* and *Dalniy Vostok* fleets killed 6,637 sperm, 245 sei, 161 fin, 40 blue, 95 Bryde's and 4 humpback whales.

ITEM 08: *Dalniy Vostok* & *Slava* whaling fleets joint Scientific Report for 1968, page 45:

Table 30. Length distribution of sperm whales (by groups) killed by the *Dalniy Vostok* and *Slava* fleets during the 1968 season.

Length (m)	<9.0	9.1-9.5	9.6-10.0	10.1-10.6	10.7-11.0	11.1-11.4	11.5-11.7	11.8-12.0	12.1-12.5	12.6-13.0	13.1-13.5	13.6-14.0	14.1-14.5	14.6-15.0	15.1-15.5	15.6-16.0	16.1-16.5	Total
females	268	373	880	2504	1391	436	410	111	4									6377
%	4.2	5.9	13.3	39.3	21.8	6.8	6.4	1.7	0.1									100
males	158	123	226	471	334	227	342	345	227	189	148	180	166	246	160	59	14	3615
%	4.4	3.4	6.2	13.1	9.2	6.3	9.4	9.6	6.3	5.2	4.1	5.0	4.6	6.8	4.4	1.6	0.4	100
Total	426	496	1106	2975	1725	663	752	456	231	189	148	180	166	246	160	59	14	9992
%	4.3	5.0	11.1	29.8	17.3	6.6	7.5	4.5	2.3	1.9	1.5	1.8	1.7	2.4	1.6	0.6	0.2	100

ITEM 09: *Dalniy Vostok* & *Slava* whaling fleets joint Scientific Report for 1969, page 105:

Table 57. Number of sperm whales caught during the 1969 season by the *Slava* and *Dalniy Vostok* whaling fleets, by size in meters.

[Length (m)]	<9.0	9.1-9.5	9.6-10.0	10.1-10.6	10.7-11.0	11.1-11.4	11.5-11.7	11.8-12.0	12.1-12.5	12.6-13.0	13.1-13.5	13.6-14.0	14.1-14.5	14.6-15.0	15.1-15.5	15.6-16.0	16.1-16.5	Total
females	397	410	857	1200	659	267	214	57	10	2								4073
%	9.8	10.0	21.4	29.4	16.1	6.5	5.2	1.4	0.25	0.05								100
males	123	82	169	272	166	124	130	153	155	138	123	158	189	308	191	74	9	2564
%	4.8	3.2	6.5	10.6	6.4	4.8	5.0	5.9	6.0	5.3	4.8	6.1	7.3	12.8	7.4	2.8	0.3	100
Total	520	492	1026	1472	825	391	344	210	165	140	123	158	189	308	191	74	9	6637
%	7.8	7.4	15.4	22.1	12.4	5.8	5.1	3.1	2.4	2.1	1.8	2.3	2.8	5.3	2.8	1.1	0.1	100

ITEM 10: Inspectors report for the whaling fleet *Vladivostok* for 1968, page 32:

Information on sperm whale size distribution during the 1968 season, by month. [Only the relevant columns used in analysis are shown; the other figures are given in the original table.]

Length (m)	July		August		September		October		November		Total	
Sex	M	F	M	F	M	F	M	F	M	F	M	F
<7.0											6	11
7.1-7.4											7	11
7.5-7.9											4	4
8.0-8.4											21	39
8.5-8.9											11	23
9.0-9.3											32	61
9.4-9.5											9	34
9.6-9.8											13	51
9.9-10.1											20	61
10.2-10.4											15	47
10.5-10.7											27	25
10.8-11.0											31	31
11.1-11.4											48	20
11.5-11.7											109	59
11.8-12.0											124	16
12.1-12.3											89	
12.4-12.6											69	
12.7-12.9											57	
13.0-13.2											58	
13.3-13.5											38	
13.6-13.8											35	
13.9-14.1											31	
14.2-14.4											47	
14.5-14.7											42	
14.8-15.0											31	

Length (m)	July		August		September		October		November		Total	
Sex	M	F	M	F	M	F	M	F	M	F	M	F
15.1-15.3											29	
15.4-15.6											14	
15.7-15.9											7	
16.0-16.3												
Total											1027	521

ITEM 11: Inspectors report for the whaling fleet *Vladivostok* for 1969, page 48:

Information on the size of killed whales for this season. [Only the relevant columns used in analysis are shown; the other figures are given in the original table.]

Length, m	Blue		Fin		Sei		Bryde's		Total	
Sex	M	F	M	F	M	F	M	F	M	F
7.1-7.4									3	4
7.5-7.7									2	9
7.8-8.0									13	34
8.1-8.3									14	31
8.4-8.6									12	43
8.7-8.9									18	35
9.0-9.3									40	136
9.4-9.5									24	104
9.6-.8									41	180
9.9-10.0									39	250
10.1-10.4									53	259
10.5-10.7									56	204
10.8-11.0									54	167
11.1-11.4									45	76
11.5-11.7									84	179
11.8-12.0									71	50
12.1-12.3									48	
12.4-12.6									47	
12.7-12.9									48	
13.0-13.2									67	
13.3-13.5									41	
13.6-13.8									54	
13.9-14.1									69	
14.2-14.4									69	
14.5-14.7									94	

Length, m	Blue		Fin		Sei		Bryde's		Total	
Sex	M	F	M	F	M	F	M	F	M	F
14.8-15.0									98	
15.1-15.3									77	
15.4-15.6									57	
15.7-15.9									37	
16.0-16.3									14	
total									1389	1757

ITEM 12: Inspectors report for the whaling fleet *Vladivostok* for 1969, page 24:

Characteristics of whaling effort by the *Vladivostok* whaling fleet for the 1967-69 seasons.

Daily catches (whales)	May	June	July	August	September	October	November	Total 1967	Total 1968	Total 1969
No catches								34	43	36
1-5								16	10	10
6-10								17	16	13
11-15								23	11	18
16-20								12	14	16
21-25								16	12	12
26-30								10	5	11
31-35								8	6	13
36-40								7	1	5
41-45								6	2	7
46-50								7	3	3
51-55								2	2	1
56-60								5	4	5
61-65								1		5
66-70								4	2	3
71-75										1
76-80										1
81-85										1
86-90										
91-95										
96-100										
Total								168	131	161

[Whaling data for Soviet fleets operating in 1973 and 1974 are identical to those contained in the IWC Catch Database, consequently the reports for these years are not translated.]

I certify that the above material (12 pages and 12 ITEMS in total) is an accurate translation of the original Russian text as appended.

Yulia Galperina-Radu
Translation Project Coordinator
HRA Inc.
6505 216th Street SW, Suite 105
Mountlake Terrace, WA 98043
USA
galperina@hrainc.net

ITEM 01

показали отсутствие промысловых скоплений китов, после чего, I6 мая флотилия направляется в юго-восточном направлении, имея целью продолжать промысел в восточном районе на скоплениях гаремных кашалотов. В восточном районе флотилия работает с I9 мая по 25 августа, добывая исключительно мелких кашалотов, в основном самок, а при подходах к северо-американскому побережью в добыче изредка встречаются усатые киты. В этом районе флотилия проработала почти половину промыслового времени и добыла более 50% общего количества китов.

Однако, к концу августа ежедневная добыча начала сокращаться и к тому же неблагоприятные метеорологические условия стали причиной ухода флотилии из Восточного района. С 26 августа по 7 сентября совершается переход в западном направлении, флотилия продвигается к району Курильских островов, где работает до I4 ноября, т.е. до конца промысла. В течение всего времени работы к/ф "Слава" в Курильском районе /от Сангарского пролива до центральной части Курильских островов/ в добыче преобладали гаремные кашалоты в большинстве самки и только в проливах Диана и Фриза несколько дней добывались крупные самцы. Усатые киты встречались редко и были представлены в основном сейвалами и единичными финвалами.

Таким образом, весь промысловый сезон I968 года к/ф "Слава" работала в основном в двух районах:

Восточном - 99 суток и Курильском - 79 суток, добыча в которых составила соответственно 37II и I484 кита.

В центральном районе, где флотилия работала непродолжительное время, и на переходах добыто I85 китов.

Общая добыча к/ф "Слава" в сезон I968 года составила 5380 китов, из которых кашалотов 5I39 /95.5%/, сейвалов

ITEM 02

скоплений китов флотилия спускается южнее и работает в Центральном районе с 6 по 30 октября, на том же контингенте китов, что и в августе. Заканчивает промысел к/ф "Дальний Восток" 10 ноября в районе центральной части Курильских островов.

В сезон 1968 года к/ф "Дальний Восток" вела промысел, главным образом, в следующих районах: в Восточном – 71 сутки, добыто 2277 китов, в Центральном – 46 суток, добыто 1829 китов, в Алеутском 23 суток, добыто 302 кита и в Командорском – 9 суток, добыто 134 кита. В Курильском районе, где флотилия работала непродолжительное время, а также на переходах добыто 160 китов.
Общая добыча к/ф "Дальний Восток" в сезон 1968 года составила 5378 китов, из которых: кашалотов 4853 /90,23%/, финвалов – 297 /5,5%/, сейвалов 154 /2,8%/, блювалов 28 /0,5%/, горбачей 24 /0,49%/, полосатиков Брайда 22 /0,48%/.

Сведения о совместной добыче китов к/ф "Дальний Восток" и "Слава" приведены в таблицах.

На прилагаемых к отчету картах дается маршрут флотилий и добыча китов по видам и по районам.

УСЛОВНЫЕ ОБОЗНАЧЕНИЯ НА КАРТАХ

_____ МАРШРУТ К/Ф „СЛАВА"

\- - - - МАРШРУТ К/Ф „ДАЛЬНИЙ ВОСТОК"

КАШАЛОТЫ ▲ от 100 до 500 ▲ от 50 до 100 ▲ до 50

● БЛЮВАЛЫ [3] СЕЙВАЛЫ +[12] БРАЙДА

(2) ФИНВАЛЫ △(1) ГОРБАЧИ

ITEM 03

Обработка материалов производилась всеми перечисленными выше сотрудниками. Для получения более полных данных по структуре стад, распределению китов и другим вопросам, в 1969г, как и в прошлых, результаты исследований полученных на 2-х базах ("Слава" и "Д.Восток") были обобщены вместе.

Настоящий отчет составлен старшими научными сотрудниками баз В.Л.Владимировым, А.А.Кузьминым и младшими научными сотрудниками баз А.Н.Николаевой, В.И.Привалихиным.

Разделы введение, промысловая обстановка, характеристика состояния стад промысловых видов китов в Центральном районе написаны А.А.Кузьминым.

Характеристика состояния стад промысловых видов китов в Восточном районе и заключение написаны В.Л.Владимировым.

Раздел характеризующий состояние сырьевой базы и состояние стад китов в Западном районе написан А.Н.Николаевой.

Обобщающий раздел, характеризующий результаты промысла и состояние сырьевой базы в северной части Тихого океана за сезон написан В.И.Привалихиным.

Промысловая обстановка

Основными районами промысла в Северной части Тихого океана в 1969 г, как и в 1968 г, служили три:

1. Восточный: 40°-50°с.ш. и 129°-160° з.д.
2. Центральный: 28°-47°с.ш.и 160°з.д.-160° в.д.
3. Курило-Японский: 34°-47°20'с.ш. и 159-173°в.д.

К/ф "Слава" начала работу 19 апреля в районе с координатами 42°22'-46°42' с.ш., 149°23'-159°48' в.д. и работала в этом районе до 24 апреля, добывая в основном кашалотов. Всего

ITEM 04

добыто 89 кашалотов и 3 финвала. С 25.4 по 9.05 работала в Центральном районе (40°10'–46°17', 163°35'–161°58' з.д.), где было добыто 313 китов; кашалотов 292, финвалов 11, блювалов 6, сейвалов 4. Однако пятнадцать дней работы в этом районе показали неэффективность ведения дальнейшего промысла и флотилия 10 мая покинула район с целью продолжать промысел в Восточном районе на скоплениях групповых кашалотов.

С 10 мая по 20 августа к/ф "Слава" работала в традиционном районе (Восточном), добывая в основном кашалотов. В течение этого периода всего добыто 2379 китов (2120 кашалотов и 259 усатых). В северной и северо-восточной акваториях района в добыче преобладали одиночки и группы кашалотов самцов. В Центральном и Восточном частях района (ближе к южной границе) флотилия работала на групповых скоплениях кашалотов (самки и маломерные самцы). Финвалы (95) в основном добывались в западной и северо-западной частях, блювалы (19) в западной и центральной; сейвалы (142) в западной и центральной частях района. Горбачи (4) добыты в восточной части района.

В отличие от прошлого года в этом отмечен более поздний (конец июня начало июля) подход групповых скоплений кашалотов. Причиной этому видимо, послужили менее благоприятные гидро и метеоусловия (среднемесячная температура воздуха и воды на глубине 8 метров в июне ниже прошлогодних, мало солнечных дней). В течение всего периода работы флотилии в Восточном районе в 1969 г. постоянно ощущалась промысловая напряженность: частые дни с проловом и большие суточные переходы обусловленные необходимостью поиска групп кашалотов, которые в этом году держались разрозненно и проявляли большую осторожность (пугливые, длительное время находились под водой, быстрое перемещение преследуе-

ITEM 05

мых животных). Китобои, как правило, вели коллективную охоту: при обнаружении китов 8–10 китобойцев устраивали охоту приемом загонов, порой выбивая всех кашалотов, не брезгуя и 7 метровыми. Такая напряженность вызвана большими научно-необоснованными, производственными планами. Несмотря на выводы и рекомендации биологических научных групп китобаз в 1968 г, которые одобрены учёным советом ТИНРО, довести план в северной части Тихого океана до 900 тыс. центнеров в год, план 1969г. и намеченный на 1970 г. остались в два раза выше.

Интенсивная промысловая нагрузка на скопления китов в Восточном районе резко отразилась на численности и биологическом состоянии Американского стада кашалотов. Продолжающееся из года в год, уменьшение численности кашалотов этого стада в сезон 1969 г создало особенно острую промысловую напряженность.

Во второй половине августа из-за снижения суточной добычи флотилия 20 августа была вынуждена покинуть Восточный район и направилась в западном направлении.

В период с 21 августа по 5 сентября флотилия "Слава" работала в западной части Центрального района добывая крупных кашалотов и единично усатых китов. Всего за это время добыто 48 кашалотов, 3 финвала и 11 сейвалов. Имея большое отставание по выполнению как годового так и квартального планов, продолжать работу в центральном районе, в виду неустойчивой суточной добычи, флотилия не могла и была вынуждена перейти в Курило-Японский район на скопления групповых кашалотов.

ITEM 06

С 6 сентября и до конца промысла к/ф "Слава" работала в районе с координатами 47°-34°с.ш. и I59°-I43°в.д. В течение всего времени работы к/ф "Слава" в Курило-Японском районе (от восточного побережья о.Хонсю до центральной части Курильских островов) в добыче преобладали групповые кашалоты в основном самки. Крупные кашалоты встречались очень редко. В сентябре, по опыту прошлого года в проливы Фриза и Диана ходил поисковый китобоец. В этом году китов в проливах не обнаружили. В водах Центральных и южных островов курильской гряды в сезон I969 г.встречались разрозненные группы кашалотов.

План сентября был выполнен на 30 %. Основное количество китов в Курило-Японском районе добыто в октябре-ноябре на восток от Хонсю и Сангарского пролива (I00-500 миль).

Весь промысловый сезон I969 г. к/ф "Слава" работала в основном в двух районах: Восточном - I0I сутки и Курильском 76 суток, добыча в которых составила соответственно 2379 и I248 китов.

В Центральном районе, где флотилия работала 28 суток, добыто 375 китов.

Общая добыча к/ф "Слава" в сезон I969 г.составила 4002 кита из них кашалотов 3704, финвалов II3, сейвалов I57, блювалов 25, горбачей 3.

К/ф "Дальний Восток" начала китовый промысел I7 июля в пределах Восточного района (46°I7' с,ш.I4I°20'з.д.). В начале промысла флотилия работала на групповых маломерных кашалотах, добывая в основном самок. По решению промыслового совета I9 июля флотилия в полном составе вышла в район с координатами

ITEM 07

добыто 65,7%.

Первую половину октября флотилия "Д.Восток" вела промысел в южной части Центрального района акватории ограниченной параллелями 31°-36° с.ш. и меридианами 170° з.д. и 170° в.д. Промысел основывался на групповых кашалотах с ограниченным выбором. В небольшом количестве добывались полосатики Брайда. Большая часть из них добыта в районе Гавайского (императорского) хребта на 34°-35°с.ш. В связи с ухудшением погоды и выполнением годового плана, в конце октября флотилия ушла из района обитания групповых кашалотов с целью продолжать промысел в водах Курильских островов на крупных кашалотов,где и работала до конца промысла. В октябре флотилией добыто 58,4 % неконвенционных кашалотов.

Несмотря на выполнение годового плана к концу октября, распоряжением начальника УКФ флотилия была задержана на промысле до 7 ноября. В ноябре флотилия работала в районе Курильских островов. Группы маломерных кашалотов встречались с океанской стороны,но охота на них не велась. За 7 дней ноября добыто всего 7 кашалотов,из них 4 маломерных самки.

За весь сезон китового промысла (с 17 июля по 6 ноября) флотилия "Д.Восток" добыла 1756 китов. Основным районом промысла был Центральный между меридианами от 160°з.д. до 160° в.д. и на юг до 31 с.ш., причем большая часть китов добыта в средней и южной частях района.

За промысловый сезон 1969 г. на к/б "Д.Восток" сдано 8 финвалов,7 блювалов,25 сейвалов и 95 полосатиков Брайда.

В 1969 г флотилиями "Слава" и "Д.Восток" добыто 6637 кашалотов,245 сейвалов, 161 финвала, 40 блювалов,95 полосатиков Брайда, 4 горбача.

ITEM 08

Таблица 30.

Размерный состав кашалотов /по группам в М/, добытых к/ф "Дальний Восток" и "Слава" в сезон I968г.

самки

	до 9,0	9,I–9,5	9,6–I0,0	I0,I–I0,6	I0,7–II,0	II,I–II,4	II,5–II,7	II,8–I2,0	I2,I–I2,5	I2,6–I3,0	I3,I–I3,5	I3,6–I4,0	I4,I–I4,5	I4,6–I5,0	I5,I–I5,5	I5,6–I6,0	I6,I–I6,5	Всего
К-во	268	373	880	2504	I39I	436	4I0	III	4									6377
%	4,2	5,9	I3,8	39,3	2I,8	6,8	6,4	I,7	0,I									I00,0

самцы

	до 9,0	9,I–9,5	9,6–I0,0	I0,I–I0,6	I0,7–II,0	II,I–II,4	II,5–II,7	II,8–I2,0	I2,I–I2,5	I2,6–I3,0	I3,I–I3,5	I3,6–I4,0	I4,I–I4,5	I4,6–I5,0	I5,I–I5,5	I5,6–I6,0	I6,I–I6,5	Всего
К-во	I58	I23	226	47I	334	227	342	345	227	I89	I48	I80	I66	246	I60	59	I4	36I5
%	4,4	3,4	6,2	I3,I	9,2	6,3	9,4	9,6	6,3	5,2	4,I	5,0	4,6	6,8	4,4	I,6	0,4	I00,0

по виду

	до 9,0	9,I–9,5	9,6–I0,0	I0,I–I0,6	I0,7–II,0	II,I–II,4	II,5–II,7	II,8–I2,0	I2,I–I2,5	I2,6–I3,0	I3,I–I3,5	I3,6–I4,0	I4,I–I4,5	I4,6–I5,0	I5,I–I5,5	I5,6–I6,0	I6,I–I6,5	Всего
К-во	426	496	II06	2975	I725	663	752	456	23I	I89	I48	I80	I66	246	I60	59	I4	9992
%	4,3	5,0	II,I	29,8	I7,3	6,6	7,5	4,5	2,3	I,9	I,5	I,8	I,7	2,4	I,6	0,6	0,2	I00,0

ITEM 09

Таблица 57.

Количество кашалотов добытых в сезон I969 г. к/б "Слава" и к/б "Дальний Восток" по размерам в метрах

САМКИ

?	до 9 м	9,I-9,5	9,6-I0,0	I0,I-I0,6	I0,7-II,0	II,I-II,4	II,5-II,7	II,8-I2,0	I2,I-I2,5	I2,6-I3,0	I3,I-I3,5	I3,6-I4,0	I4,I-I4,5	I4,6-I5,0	I5,I-I5,5	I5,6-I6,0	I6,I-I6,5	Всего
Кол-во	397	4I0	857	I200	659	267	2I4	57	I0	2	2	-	-	-	-	-	-	4073
%	9,8	I0,0	2I,4	29,4	I6,I	6,5	5,2	I,4	0,25	0,05	-	-	-	-	-	-	-	I00

САМЦЫ

Колуво	I23	82	I69	272	I66	I24	I30	I53	I55	I38	I23	I58	I89	308	I9I	74	9	2564
%	4,8	3,2	6,5	I0,6	6,4	4,8	5,0	5,9	6,0	5,3	4,8	6,I	7,3	I2,8	7,4	2,8	0,3	I00

ПО ВИДУ

Кол-во	520	492	I026	I472	825	39I	344	2I0	I65	I40	I23	I58	I89	308	I9I	74	9	6637
%	7,8	7,4	I5,4	22,I	I2,4	5,8	5,I	3,I	2,4	2,I	I,8	2,3	2,8	5,3	2,8	I,I	0,I	I00

ITEM 10

СВЕДЕНИЯ
О РАЗМЕРАХ КАШАЛОТОВ ЗА 1968 ГОД ПОМЕСЯЧНО

[illegible]	июль		август		сентябрь		Октябрь		Ноябрь		ИТОГО	
	о-цм	о-км	о-цм	о-км	о-цм	о-км	о-цм	о-км	о-цм	о-км	о-цм	о-км
7,0							6	II			6	II
-7,4							7	II			7	II
-7,9							4	4			4	4
-8,4			6	IO			I5	29			2I	39
-8,9		I	2	3			9	I9			II	23
-9,3	I		4	4			27	77			32	8I
-9,5			2	3			7	3I			9	34
-9,8		2	2	3			II	46			I3	5I
-I0,I	I	I	7	3			I2	57			20	6I
-I0,4		I	3	4			I2	42			I5	47
-I0,7	5		I0	4			I2	2I			27	25
-II,0	9	2	I8	5			4	24			3I	3I
-II,4	I3	4	25	I0			I0	I4			48	28
-II,7	27	I4	4I	22			4I	23			I09	59
3-I2,0	4I	2	48	9	3		30	5	2		I24	I6
I-I2,3	24		4I		7		4		3		89	
4-I2,6	22		35		8		2		2		69	
7-I2,9	2I		26		7		2		I		57	
0-I3,2	24		I5		I9						58	
3-I3,5	20		II		6				I		38	
6-I3,8	I2		I5		I0				I		38	
9-I4,I	I6		I2		3						3I	
2-I4,4	I4		28		4				I		47	
5-I4,7	I9		I8		3				2		42	
8-I5,0	I5		I3		3						3I	
I-I5,3	II		I4		3				I		29	
4-I5,6	7		6		I						I4	
7-I5,9	2		3		I				I		7	
0-I6,5												
[illegible]ГО:	3I4	27	405	80	78		2I5	4I4	I5		I087	52I

ITEM 11

СВЕДЕНИЯ О РАЗМЕРАХ КИТОВ ЗА ГОД

Длина в метрах	Количество китов									
	Блювалы		Финвалы		Сейвалы		Брайда		Кашалоты	
	♂	♀	♂	♀	♂	♀	♂	♀	♂	♀
7,0-7,4									3	4
7,5-7,7									2	9
7,8-8,0									I3	24
8,I-8,3									I4	3I
8,4-8,6									I2	43
8,7-8,9									I8	35
9,0-9,2									40	I36
9,3-9,5									24	I04
9,6-9,8									4I	I80
9,9-I0,0									39	250
I0,I-I0,4									58	258
I0,5-I0,7									56	204
I0,8-II,0					I			I	54	I67
II,I-II,4									45	78
II,5-II,7								I	84	I79
II,8-I2,0						I			7I	50
I2,I-I2,3					I5	I0	I	I	48	
I2,4-I2,6					I0	2I		2	47	
I2,7-I2,9									48	
I3,0-I3,2					I7	II		5	67	
I3,3-I3,5					8	I4			4I	
I3,6-I3,8									54	
I3,9-I4,I			I	I	7	I6			59	
I4,2-I4,4									69	
I4,5-I4,7					2	I3			94	
I4,8-I5,0						I2			98	
I5,I-I5,3									77	
I5,4-I5,6					I	I			57	
I5,7-I5,9									37	
I6,0-I6,3						I			I4	
I6,4-I6,6		I			I					
I6,7-I6,9			2	6						
I7,0-I7,2			6	3						
I7,3-I7,5		I	3	4						
I7,6-I7,8			I							
I7,9-I8,I			6	3						
I8,2-I8,4										
I8,5-I8,7			3	7						
I8,8-I9,0	I	2	4	5						
I9,I-I9,3										
I9,4-I9,6		I		I						
I9,7-I9,9				I						
20,0-20,2				I						
20,3-20,5			2	I						
20,6-20,8									II,8м-	II,5м
20,9-2I,I		4	I	I					-I0г	-I5г
2I,2-2I,4										
2I,5-2I,7	4	I		2						
2I,8-22,0	I									
22,I-22,3										
22,4-22,6	I	I								
22,7-22,9				I						
23,0-23,3		2								
ИТОГО:	7	I2	29	37	62	I0I	I	I0	I389	I757

За период промысла были добыты также один горбач и один гладкий кит.

ITEM 12

Характеристика интенсивности промысла
по к/б "Владивосток" за 1967-69 гг.

Суточная добыча (гол)	Май 67г.	Май 68г.	Май 69г.	Июнь 67г.	Июнь 68г.	Июнь 69г.	Июль 67г.	Июль 68г.	Июль 69г.	Август 67г.	Август 68г.	Август 69г.	Сентябрь 67г.	Сентябрь 68г.	Сентябрь 69г.	Октябрь 67г.	Октябрь 68г.	Октябрь 69г.	Ноябрь 67г.	Ноябрь 68г.	Ноябрь 69г.	Итого 67г.	Итого 68г.	Итого 69г.
Добычи нет	4			3		3	9	4	3	2	7	8	5	7	5	8	9	II	9	I6	6	34	43	36
1-5	3						5	3		3		I	4	2	3	I	5	5	I		I	I6	I0	I0
6-10	3					I	I		2	6	7	3	4	5	4	3	2	3	I	2		I7	I6	I3
11-15	5			I		3	3	2	2	7	4	4	5	5	6	2		3	4			23	II	I8
16-20	2			5			I	4	5	3	2	7		4		I	4	4	I			I2	I4	I6
21-25	I			4		I	4	3	4	5	4	4	I	4	3	I	I					I6	I2	I2
26-30				I		2	I	2	4		2	2	6			2	I	2	3		I	I0	5	II
31-35				2		4	3	2	5	2	2	I	I	2				3				8	6	I3
36-40	I			3		3			I	I	I				I	2						7	I	5
41-45				4		2					I				4		I		2		I	6	2	7
46-50				3		I	2			I		I		I	I	I	2					7	3	3
51-55				I		I	I										2					2	2	I
56-60				2		2	I		2	I	I				I	I	3					3	4	5
61-65						3			I				I		I				I			I		5
66-70				I		I		I	2							3	I					4	2	3
71-75															I									I
76-80						I																		I
81-85						I																		I
86-90																								
91-95																								
96-100																								
ИТОГО:	I9			30		29	3I	3I	3I	3I	3I	3I	30	30	30	27	3I	3I	22	I8	9	I68	I3I	I6I
